# A PacBio single molecule real-time sequencing-based full-length transcriptome atlas of tree tomato (*Solanum betaceum* Cav.) and mining of simple sequence repeat markers

**DOI:** 10.3389/fpls.2022.1052817

**Published:** 2022-11-02

**Authors:** Honghong Deng, Lu Zhang, Ming’an Liao, Jin Wang, Dong Liang, Hui Xia, Xiulan Lv, Qunxian Deng, Xun Wang, Yi Tang, Lijin Lin

**Affiliations:** ^1^ Institute of Pomology and Olericulture, Sichuan Agricultural University, Chengdu, China; ^2^ College of Horticulture, Sichuan Agricultural University, Chengdu, China

**Keywords:** tree tomato, PacBio single molecule real-time sequencing, full-length transcriptome, simple sequence repeat, molecular marker

## Introduction

Tree tomato (*Solanum betaceum* Cav., syn. *Cyphomandra betacea* (Cav.) Sendtn.), or tamarillo, is a fast-growing fruit species of the family *Solanaceae* genus *Solanum* (Acosta-Quezada et al., 2015). Native to the Andean region of South America, tree tomato cultivation has spread to several countries of the tropics and subtropics (South America, New Zealand, Australia and India) (Ramírez and Kallarackal, 2019). Recently, tree tomato has received increasing attention, as a rich source of sugars, organic acids, minerals, vitamins (vitamin C and B_6_), carotenoids, anthocyanins, and phenolics (Acosta-Quezada et al., 2015; Espin et al., 2016). Thus, it developed from a disregarded crop to a promising fruit crop (Pacheco et al., 2021).

Previous studies on tree tomato mainly focused on its biochemical properties (Acosta-Quezada et al., 2015; Espin et al., 2016), phenology (Acosta-Quezada et al., 2016), and reproductive biology, including flower and pollen morphology, physiology, fruit characteristics, intraspecific hybridization, and genetic diversity (Ramírez and Kallarackal, 2019). Despite the recent interest and research progress, reference genome and transcriptome of tree tomato are not available, which hampers in-depth functional genomics, molecular genetics and genetic-assisted breeding. Additionally, *de novo* assembly of transcriptome sequence through old-fashioned second-generation short-read sequencing, has been challenging, without a well-annotated reference genome (Amarasinghe et al., 2020). The advent of PacBio long-read single-molecule real-time (SMRT) sequencing approach addressed these challenges and provided the opportunity to obtain reliable genome-wide full-length (FL) transcripts directly (Amarasinghe et al., 2020).

Transcriptome profiling has proved an effective approach for the genome-wide development of simple sequence repeat (SSR) markers in several non-model plants, at a large scale and low cost (Deng et al., 2018; Jia et al., 2020). SSRs, used for genetic mapping, serve as DNA fingerprinting markers to assess genetic diversity and population structure. Furthermore, SSRs can be useful to distinguish closely-related cultivars, due to their advantages of single locus, multiple allele variations, and abundant polymorphism (Liu et al., 2015). To date, only amplified fragment length polymorphism (AFLP) markers were used to evaluate the genetic diversity between different tree tomato varieties (Acosta-Quezada et al., 2012). Therefore, identification of SSR markers at genome-wide scale for tree tomato are highly desirable.

In this study, we constructed for the first time, to the best of our knowledge, an atlas of tree tomato’s FL transcriptome and analysed the distribution of SSR motifs.

## Value of the data

- Using PcaBio SMRT sequencing, we constructed for the first time, an atlas of the FL transcriptome of tree tomato. This will facilitate further study of genome annotation to this crop, opening an exciting avenue in transcriptome-based studies, such as posttranscriptional regulation events analyses.
- To the best of our knowledge, no SSR markers were available for tree tomato gene mapping, until now. The current study encompasses the first mining and development of SSR markers in tree tomato, which will be determinant for genetic studies and molecular marker-assisted breeding in this fruit crop.

## Materials and methods

### Plant materials

Five-year-old plants of tree tomato were grown at the experimental base of the College of Horticulture, Sichuan Agricultural University, Chengdu, China (30.71˚N, 103.87˚E). Seven tissues (root tips, shoot tips, mature leaves, flower buds, flowers in full bloom, young fruit, and mature fruit) of three independent mature trees, and three tissues (root tips, shoot tips, and leaves) of three seedlings were sampled and mixed. Seedlings were obtained by incubation of seeds at 22˚C and 95% relative humidity.

### Library preparation and PacBio sequencing

Total RNA was extracted using a PureLink RNA mini kit (Invitrogen, CA, USA), followed by DNase digestion and RNA purification using an on-column PureLink DNase kit (Invitrogen). RNA concentration and purity were determined using a NanoPhotometer spectrophotometer (Implen, CA, USA). RNA integrity was determined using an RNA Nano 6000 assay kit on a Bioanalyzer 2100 system (Agilent Technologies, CA, USA). RNA integrity number (RIN) > 7.0 and 2.0 < OD 260/280 < 2.2 were the RNA quality requirements for the RNA samples. Iso-Seq cDNA library was constructed and PacBio sequencing were performed at Novogene Co., Ltd. (Beijing, China). The mRNA was enriched using oligo-dT magnetic beads in 4.0 μg total RNA and reverse transcribed into cDNA using the SMARTer PCR cDNA synthesis kit (Clontech, now Takara, http://www.takarabio.com). The size-selected cDNA library was constructed according to the BluePippin size selection system (Sage Science, MA, USA) protocol and sequenced on the PacBio sequel platform.

### Reads processing and error collection

Raw data were processed using SMRTlink v5.0 software. Circular consensus sequencing (CCS) reads were yielded from subread Binary Alignment Map (BAM) files. The full-length non-chimeric (FLNC) reads and non-full-length reads were determined by the simultaneous presence of the poly-A tail signal and the 5’ and 3’ cDNA primers from reads of insert (ROIs). Short reads (shorter than 50 bp in length) were discarded. FLNC sequences were isoform-level clustered with iterative clustering and error correction (ICE) software, generating one consensus isoform (Gordon et al., 2015). The non-full-length CCSs were polished using the Quiver algorithm. High quality FL transcripts were defined with the criterial of//a minimum Quiver accuracy of 0.99.

### Functional annotation and transcript analysis

Gene functional annotation was performed using the National Center for Biotechnology Information (NCBI) non-redundant protein (Nr, E-value ≤1 e-5), NCBI non-redundant nucleotide (Nt, E-value ≤1 e-5), gene ontology (GO, E-value ≤1e-10), Kyoto encyclopedia of genes and genomes (KEGG, E-value ≤1e-3), eukaryotic orthologous groups (KOG, E-value ≤1e-3), Swissprot protein (E-value ≤1e-5^-5^), and protein family (Pfam, E-value ≤0.01) databases.

Coding sequence (CDS) was predicted by ANGEL (Robust Open Reading Frame prediction) with default parameters (Shimizu et al., 2006). Transcription factors (TFs) were predicted using iTAK software ((http://itak.feilab.net/cgi-bin/itak/index.cgi) (Zheng et al., 2016). Long non-coding RNA (LncRNA) was firstly screened *via* coding-non-coding-index with default parameters (Kong et al., 2007) and Coding Potential Calculator with NCBI eukaryotes’ protein database (E-value <1e-10) (Sun et al., 2013). Each transcript was then translated in three possible frames, and Pfam Scan with default parameters of -E 0.001 –domE 0.001 was employed to determine the existene of a known protein family domain. SSRs were identified by MISA program (https://pgrc.ipk-gatersleben.de/misa/).

## Results

### Full-length transcriptome of tree tomato

A total of 9.92G subreads base was obtained, comprising 9,877,631 subreads, with an average subreads length of 1,005 bp and an N50 length of 1,974 bp. Approximately 70.41% of the subreads fell within the size range of 200 to 1,000 bp. Of the 416,144 CCS isoforms, 308,699 were identified as consensus FLNC reads, with a mean length of 2,099 bp (**Table 1**).

**Table 1:** Summary of circular consensus sequence of tree tomato (*Solanum betaceum* Cav.) generated by SMRT sequencing technology.

Sample	CCS	5’-primer	3’-primer	Poly-A	Full length	FLNC	Average FLNC read length	Consensus reads
*betacea*	416144	372441	381814	378908	322600	308699	2099	167191

**Figure 1:**
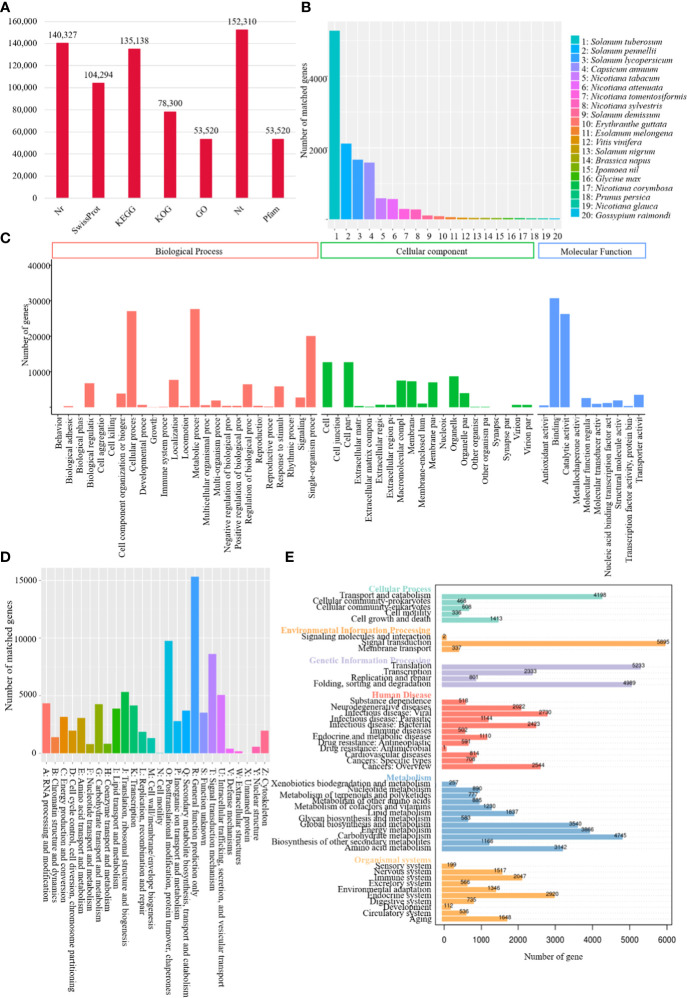
Functional annotation of tree tomato full-length transcriptome. **(A)** Bar chart demonstrating tree tomato annotated number of genes in different databases; **(B)** homologous species distribution of tree tomato transcripts annotated in Nr database; **(C)** distribution of GO terms for all tree tomato annotated transcripts; **(D)** KOG classification of tree tomato transcripts; **(E)** KEGG enrichment of tree tomato transcripts.

A total of 140,327, 104,294, 135,138, 78,300, 53,520, 152,310 and 53,520 transcripts were functionally annotated by sequence similarity search against Nr, Swiss-Prot, KEGG, KOG, GO, Nt and Pfam databases, respectively (**Figure 1A**). Annotation of Nr homologous species distribution showed the best blast hit with tree tomato and *Solanum tuberosum* (52,712 isoforms), *Solanum pennellii* (21,171 isoforms), *Solanum lycopersicum* (16,666 isoforms), and *Capsicum annuum* (15,851 isoforms) (**Figure 1B**). Transcripts were classified into three GO categories, including biological process, cellular component, and molecular function, in which the most abundant subcategory was metabolic process (27,699 matched genes, 51.75%), cell (12,693 matched genes, 23.72%), and binding (30,712 matched genes, 57.38%), respectively (**Figure 1C**).

KOG analysis showed tree tomato transcripts were assignmented to a total of 26 categories. The largest group belonged to general function prediction only (15,323 matched genes, 19.57%), followed by posttranslational modification, protein turnover, chaperones (9,750, 2.45%) and signal transduction mechanism (8,614, 11.00%) (**Figure 1D**). KEGG functional classification showed a total of 5895 out of the 135,138 transcripts assigned to the signal transduction, thus making it the largest group (4.36%) amongst the major categories, followed by translation (5,233, 3.87%), folding, sorting and degradation (4,989, 3.69%), and carbohydrate metabolism (4,745, 3.51%) (**Figure 1E**).

### Structure analysis and SSR identification

**Figure 2:**
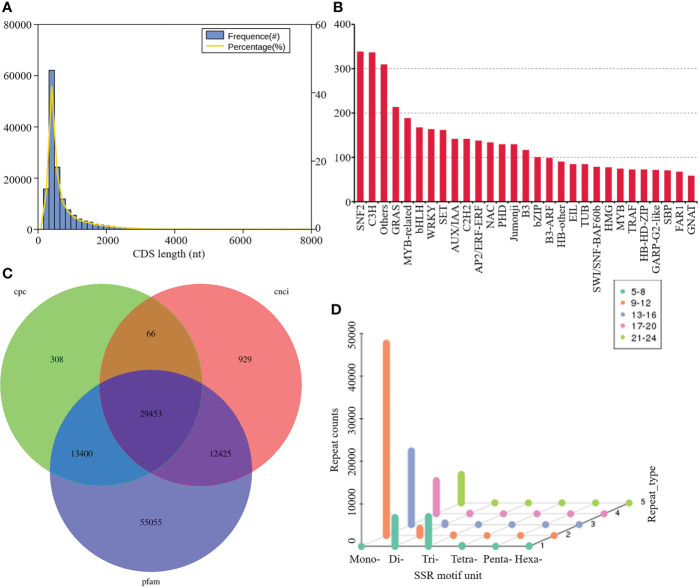
Structure Analysis and SSR Identification. **(A)** Frequency histogram depicting length distribution of CDS; **(B)** distribution of TFs; **(C)** Venn diagram summarizing lncRNAs number; **(D)** SSR motifs frequency.

The frequencies for each length of CDS were evaluated with the most prevalent length of CDS ranged from 400 to 2,000 bp (**Figure 2A**). A total of 5,114 genes were predicted to be TFs belonging to different families, amongst which the most abundant was SNF2 (338 matched genes, 6.61%), followed by C3H (336, 6.57%), others (309, 6.04%), GRAS (213, 4.17%), MYB-related (188, 3.68%), bHLH (167, 3.27%), WRKY (163, 3.19%), and SET (161, 3.15%) (**Figure 2B**). A total of 43227, 42872, and 110333 noncoding RNAs candidates were predicted by CPC, CNCI, and Pfam databases, respectively. Among them, 29,453 transcripts were simultaneously identified by the three computational approaches (**Figure 2C**). A screen of the 79549 transcripts using MISA program yielded diverse SSR types, including mononucleotide, dinucleotide, trinucleotide, tetranucleotide, pentanucleotide, hexanucleotide, and some complex nucleotides. Amongst these, mononucleotide repeats (63.97%) exhibited the highest frequency of occurrence, followed by dinucleotide (8.54%) and trinucleotide repeats (7.79%) (**Figure 2D**).

## Data availability statement

The original contributions presented in the study are publicly available. This data can be found here: NCBI, PRJNA883812 (https://www.ncbi.nlm.nih.gov/bioproject/PRJNA883812). The function annotation, gene structure analysis and SSR identification have been deposited at the Figshare database with doi: 10.6084/m9.figshare.21200887.

## Author contributions

LL conceived the idea and acquired funding; LZ, ML, JW, DL, and HX collected the samples and conducted the experiment; HD, XL, QD, XW, YT, and LL performed analysis on the data; HD wrote and revised the manuscript. All authors contributed to the article and approved the submitted version.

## Funding

This work was funded by the “Shuangzhi” program of Sichuan Agricultural University.

## Conflict of interest

The authors declare that the research was conducted in the absence of any commercial or financial relationships that could be construed as a potential conflict of interest.

## Publisher’s note

All claims expressed in this article are solely those of the authors and do not necessarily represent those of their affiliated organizations, or those of the publisher, the editors and the reviewers. Any product that may be evaluated in this article, or claim that may be made by its manufacturer, is not guaranteed or endorsed by the publisher.
